# Influences of specific ions in groundwater on concrete degradation in subsurface engineered barrier system

**DOI:** 10.1186/s40064-016-2495-8

**Published:** 2016-06-16

**Authors:** Wen-Sheng Lin, Chen-Wuing Liu, Ming-Hsu Li

**Affiliations:** Hydrotech Research Institute, National Taiwan University, Taipei City, Taiwan; Department of Bioenvironmental Systems Engineering, National Taiwan University, Taipei City, Taiwan; Institute of Hydrological and Oceanic Sciences, National Central University, Taoyuan City, Taiwan

**Keywords:** Concrete degradation, Hydrogeochemistry, Reactive chemical transport, Engineered barriers system, Radioactive waste disposal

## Abstract

Many disposal concepts currently show that concrete is an effective confinement material used in engineered barrier systems (EBS) at a number of low-level radioactive waste (LLW) disposal sites. Cement-based materials have properties for the encapsulation, isolation, or retardation of a variety of hazardous contaminants. The reactive chemical transport model of HYDROGEOCHEM 5.0 was applied to simulate the effect of hydrogeochemical processes on concrete barrier degradation in an EBS which has been proposed to use in the LLW disposal site in Taiwan. The simulated results indicated that the main processes that are responsible for concrete degradation are the species induced from hydrogen ion, sulfate, and chloride. The EBS with the side ditch drainage system effectively discharges the infiltrated water and lowers the solute concentrations that may induce concrete degradation. The redox processes markedly influence the formations of the degradation materials. The reductive environment in the EBS reduces the formation of ettringite in concrete degradation processes. Moreover, the chemical conditions in the concrete barriers maintain an alkaline condition after 300 years in the proposed LLW repository. This study provides a detailed picture of the long-term evolution of the hydrogeochemical environment in the proposed LLW disposal site in Taiwan.

## Background

The engineered barriers system (EBS) is an integral part of the radioactive waste disposal facility. The EBS represents the manmade, engineered materials of a repository, including the waste form, waste canisters, concrete barrier, buffer materials, backfill, and seals, and can be used as physical and/or chemical obstructions to prevent or hinder the migration of radionuclides (Dupray and Laloui 2014; IAEA 2001a). The function of the EBS is to prevent and/or hinder the release of radionuclides from the waste to host rock and biosphere. However, the lack of an appropriate EBS design in the concrete barrier, backfill, and the selection of sealing and covering materials for trenches, vaults, and ditches may result in the ingress of groundwater and the release of radionuclides from the disposed wastes (IAEA 1999, 2001a, b).

Several current disposal concepts indicate that concrete is an effective confinement material that is used in engineered barriers at a number of low-level radioactive waste (LLW) disposal sites in most countries (IAEA 2001a, b). Over the past few decades, a number of studies focused on the assessment of concrete barriers with the properties of encapsulation, isolation, or retardation of a variety of nuclear hazardous contaminants to ensure the reliable long-term performance of such a disposal concept. Jantzen et al. (2010) proposed the use of cements in radioactive and hazardous waste disposal and provided an advanced understanding of cement–waste interactions and the mechanisms in which contaminants are retained. Glasser et al. (2008) discussed cement paste deterioration by detrimental chemical reactions and the mechanisms that manage the transport of ions, moisture, and gas. He also reviewed various chemical degradation phenomena, such as microstructural alterations, that result from exposure to chlorides and carbon dioxide and sulfate attacks from external sources that result in the formation of ettringite and thaumasite. However, a major concern of this EBS design is that concrete may be subject to degradation by re-crystallization and chemical reactions with the aqueous environment. A number of reactions may occur simultaneously in groundwater and cement-based materials, including the dissolution of portlandite that is generated from the intrusion of hydrogen ion, the increase of the concentration of calcium in the pore solution, formation of ettringite by sulfate attacking the cement, and the dissolution of Calcium–Silicate–Hydrate (CSH) gel by chloride entering the cement, and the formation of Friedel’s salt (Luna et al. 2006). Moreover, Bruno et al. (1999) indicated that the interaction of pore water with accessory minerals of bentonite, such as sulfate dissolution-precipitation, and pyrite oxidation, controlled the geochemical characteristics of the system. The repository environment (for example, the pH, Eh, and ionic composition of the surrounding water) affects EBS performance in waste storage and disposal. By providing adequate redox conditions that inhibit sulfate-containing water to reduce the sulfate attack on cement and concrete may facilitate the expected design performance of the concrete barrier. However, few studies have indicated that the conversion of sulfide to sulfate concomitantly occurs with pyrite oxidation and the accompanying creation of a reductive environment in the EBS to reduce the ettringite formation. Limited attention has been focused on redox processes that may markedly influence the formations of degradation materials from concrete (Berner 1992). Therefore, concrete degradation that is caused by the altered redox environment requires further investigation.

The hydrogeochemical environment of an LLW repository is determined by the composition of groundwater and mineral formation, which may influence the chemical compatibility of backfill material, the concrete barrier, and the buffer material in the near field. The durability of cementitious material in service environments has presented a number of concerns, such as whether the EBS may be completely isolated from the groundwater and the accompanying hydrogeochemical reactions and key aqueous species in the groundwater that affect the degradation of the concrete barrier of the repository, and the influence of the redox processes on the formations of degradation materials. To obtain further insights into these interactions and provide a detailed overview of the long-term evolution in the hydrogeochemical environment of the concrete barrier, a reactive chemical transport model of HYDROGEOCHEM 5.0 (Yeh et al. 2004) was used to assess the hydrogeochemical influences on concrete barrier degradation. The results of this study offer valuable information on the long-term behavior of the concrete barrier in the EBS.

## Study area

### Site description

**Fig. 1:**
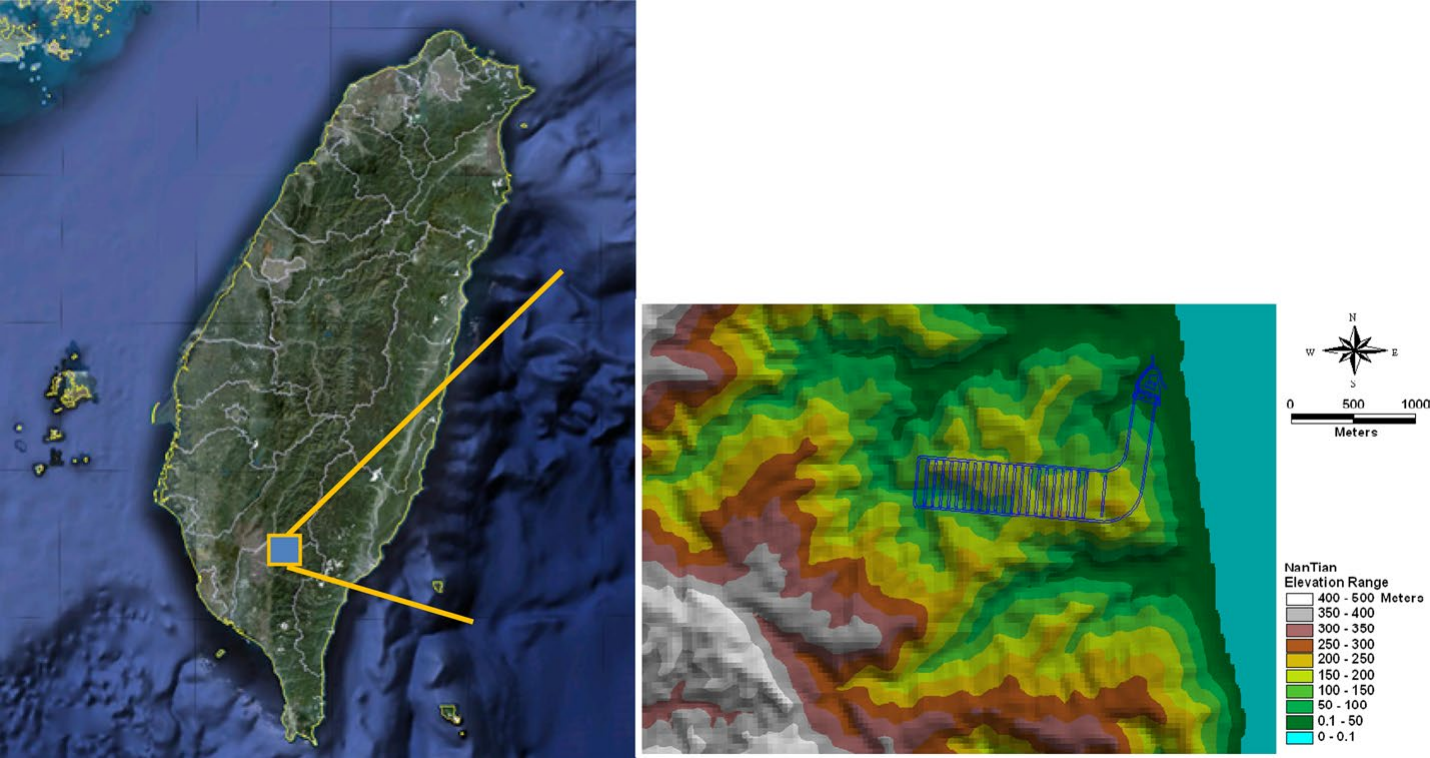
The location of the proposed site in Taiwan (Google earth)

In Taiwan, LLW is generated from a variety of commercial, medical, industrial, and educational activities. The site selection for LLW disposal is a challenging task. The general concerns include the hydrological and hydrogeological site conditions. A proposed site for the final disposal of LLW is located in the Daren Township of Taitung County along the southeastern coast of Taiwan as shown in Fig. 1. The geology of the Daren site consists of argillite and meta-sedimentary rocks. A mined cavern design with a tunnel system of 500 m below the surface is proposed. Concrete is used as the confinement material for the engineered barrier (Taiwan Power Company 2010).

### Concrete barrier design

**Fig. 2:**
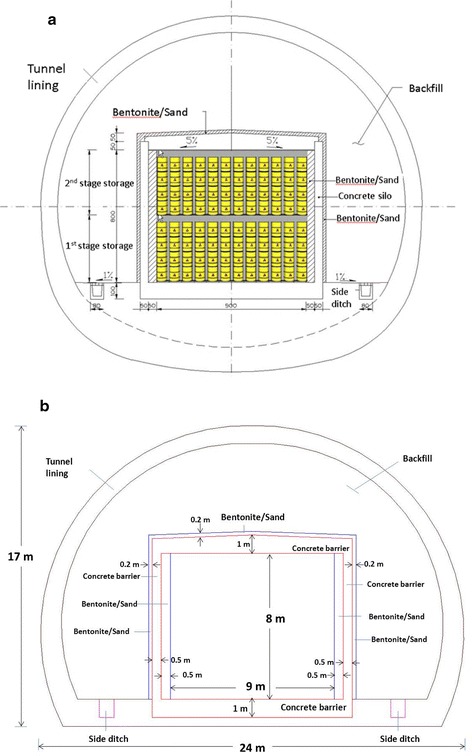
Schematic illustration of the EBS design in the proposed site: **a** cross-section of the tunnel, **b** conceptual model for the cross-section of the tunnel

Concrete is mainly composed of a mixture of cement, water, and aggregates. The aggregates consist of minerals such as sand, gravel, and stone, which are chemically inert to water and concrete solid. The mineral compounds in cement are calcium silicates (Ca_3_SiO_5_ and Ca_2_SiO_4_), aluminate (Ca_3_Al_2_O_6_), and ferrite (4CaO·Al_2_O_3_·Fe_2_O_3_), abbreviated as C3S, C2S, C3A, and C4AF, respectively. When these constituents are mixed with water, calcium–silicate–hydrate (C–S–H), portlandite (Ca(OH)_2_(s)), ettringite (Aft), monosulfate (AFm), and hydrogarnet form in the cement hydration process. These hydration products also control the setting and hardening of the concrete. These alterations have direct consequences on the engineering properties of the concrete barrier. Figure 2 schematically illustrates the design of an engineered barrier in the proposed Daren site. The thickness of the concrete barrier ranges from 0.5 to 1.0 m. The inner and outer thicknesses of the bentonite buffer are 0.5 and 0.2 m, respectively.

The chemical composition of Portland Type I Cement for Taiwan Cement Company and the calculated amount of the clinker phases in the unhydrated cement that was used in this study are listed in Table 1. The mixed proportion of concrete components and their physical properties are summarized in Table 2.

**Table 1:** Chemical composition and corresponding clinker phases in Taiwan cement

Component	Content % by weight
CaO	61.96
SiO_2_	20.42
Al_2_O_3_	4.95
Fe_2_O_3_	3.09
MgO	3.29
Na_2_O + 0.685K_2_O	Max. 0.6
SO_3_	2.4
Corresponding clinker component	
Tricalcium silicate (C_3_S)	49
Dicalcium silicate (C_2_S)	21
Tricalcium aluminate (C_3_A)	7.9
Tetracalcium aluminate ferrite (C_4_AF)	9.4

Abbreviations used as following: C, CaO; S, SiO_2_; A, Al_2_O_3_; F, Fe_2_O_3_; H, H_2_O

**Table 2:** Mixed proportion of concrete components and their physical properties

Component	Content % by volume
Cement + Water	30
Ballast	70

Physical properties	Parameter
Density	2030 kg/m^3^
w/c ratio	0.5
Porosity	0.15

### Numerical models

The groundwater quality data was limited because a groundwater monitoring well was not set up in the proposed site. Therefore, a thermodynamic equilibrium model, PHREEQCI (USGS 2010), was used to determine the equilibrium composition of the pore water as groundwater reacting with geological mineral of argillite at the site. The calculated results represented the groundwater quality of the proposed site in this study.

To evaluate the durability of the cementitious materials of the concrete barrier in the near field, a multi-species coupled geochemical transport model HYDROGEOCHEM 5.0 was used to simulate the reactive geochemical transport processes that are involved in the concrete degradation.

### Chemical equilibrium model

The PHREEQCI is a complete windows-based graphical user interface version of the geochemical computer program PHREEQC (Parkhurst and Appelo 1999) which was developed by the U.S. Geological Survey, and provides all of the capabilities of the geochemical model PHREEQC, including speciation, batch-reaction, 1D reactive-transport, and inverse modeling.

### Reactive chemical transport model

To assess the durability of the cementitious materials of the concrete barrier, a hydrogeochemical transport model HYDROGEOCHEM 5.0 was used to simulate the reactive chemical transport processes that are involved in the concrete degradation in the near field. The computer program HYDROGEOCHEM 5.0, developed by Yeh et al. (2001, 2004), is a 3-D numerical model of fluid flow, thermal, hydrologic transport, and biogeochemical kinetic and equilibrium reactions in saturated and unsaturated media. HYDROGEOCHEM 5.0 was designed for generic applications to reactive transport problems that are controlled by both kinetic and equilibrium reactions in subsurface media. The flow equations, chemical transport equations, chemical equilibrium equations, initial boundary conditions, and numerical methods of the model are described as follows.

#### Flow equations

A modified Richard’s equation describes the density-dependent fluid flow in variably-saturated media. It is derived based on the continuity of fluid, continuity of solid, motion of fluid (Darcy’s law), consolidation of the media, and the compressibility of water, as follows:

1 $$\frac{\rho }{{\rho_{0} }}F\frac{\partial h}{\partial t} = \nabla \cdot \left[ {{\mathbf{K}} \cdot \left( {\nabla h + \frac{\rho }{{\rho_{0} }}\nabla z} \right) + \frac{{\rho^{*} }}{{\rho_{0} }}q} \right]$$

2 $$F = a^{{\prime }} \frac{\theta }{{n_{e} }} + \beta^{{\prime }} \theta + n_{e} \frac{dS}{dh}$$

3 $${\mathbf{V}} = - {\mathbf{K}} \cdot \left( {\frac{{\rho_{0} }}{\rho }\nabla h + \nabla z} \right)$$

where *θ* is the effective moisture content (L^3^/L^3^), *h* is the pressure head (L), *t* is the time (T), *z* is the potential head (L), *q* is the source or sink representing the artificial injection or withdrawal of fluid [(L^3^/L^3^)/T], *ρ*_0_ is the referenced fluid density at zero biogeochemical concentration (M/L^3^), *ρ* is the fluid density with dissolved biogeochemical concentrations (M/L3), ρ* is the fluid density of either injection (=ρ*) or withdraw (=*ρ*) (M/L^3^), ${\mathbf{K}}$ is the hydraulic conductivity tensor (L/T), *α*′ is the modified compressibility of the soil matrix (1/L), *β*′ is the modified compressibility of the liquid (1/L), *n*_*e*_ is the effective porosity (L3/L3), and *S* is the degree of effective saturation of water.

The finite element method was used to solve Eqs. (1), (2), and (3), and the constitutive relationships among the pressure head, degree of saturation, and hydraulic conductivity tensor, together with the appropriate initial conditions and the five types of boundary conditions, which are Dirichlet, Cauchy, Neumann, variable, and surface-water boundary conditions. The temporal-spatial distributions of the hydrological variables, including pressure head, total head, effective moisture content, and Darcy’s velocity were obtained.

#### Chemical transport equations

The governing equations for transport were derived based on the continuity of mass and Fick’s flux laws. The main transport and fate processes are advection, dispersion/diffusion, source/sink, and biogeochemical reactions (including radioactive decay). The general transport equation governing the temporal-spatial distribution of any biogeochemical species in a reactive system is described below. Let *C*_*i*_ be the concentration of the *i*th species, the governing equation for *C*_*i*_ was obtained by applying the principle of mass balance in integral form as follows:

4 $$\frac{{\partial \theta C_{i} }}{\partial t} + \theta \alpha^{\prime} \frac{\partial h}{\partial t}C_{i} = L(C_{i} ) + \theta r_{i} + M_{i} \varvec{,}\quad i \in \left\{ M \right\}$$

where *L* is the transport operator denoting

5 $$L(C_{i} ) = - \nabla \cdot ({\textbf{V}}C_{i} ) + \nabla \cdot \left[ {\theta {\textbf{D}} \cdot \nabla C_{i} } \right]$$

where *C*_*i*_ is the concentration of the *i*-th species in units of chemical mass per water volume [M/L^3^]; *r*_*i*_ is the production rate of the *i*-th species because of biogeochemical reactions in chemical mass per water volume per unit time [M/L^3^/T]; $\left\{ {\text{M}} \right\} = \left\{ {1,2, \ldots ,{\text{M}}} \right\}$ in which *M* is the number of biogeochemical species; **D** is the dispersion coefficient tensor [L^2^/T]; and *M*_*i*_ is the source/sink (other than sources because of chemical reactions) of the *i*-th species in chemical mass per unit volume of media [M/L^3^/T].

Four types of boundary conditions (Dirichlet, Cauchy, Neumann, and variable inflow-outflow) were implemented in HYDROGEOCHEM5.0.

#### Chemical reaction equations

The formulation of rate equations for all N reactions is the crucial factor in modeling mixed equilibrium and geochemical kinetic reactions. A rate equation must be specified to quantitatively describe a general geochemical reaction that is written as:

6 $$\sum\limits_{{i \in \left\{ M \right\}}} {\mu_{ik} \mathop {C_{i} }\limits^{ \wedge } } \leftrightarrow \sum\limits_{{i \in \left\{ M \right\}}} {\nu_{ik} \mathop {C_{i} }\limits^{ \wedge } } \varvec{,}\quad k \in \left\{ N \right\}$$

where $\mathop {C_{i} }\limits^{ \wedge }$ is the chemical formula of the *i*th species; *μ*_*ik*_ is the reaction stoichiometry of the *i*th species in the *k*th reaction associated with the reactants; *ν*_*ik*_ is the reaction stoichiometry of the *i*th species in the *k*th reaction associated with the products; {*N*} = {1, 2, …, *N*} in which *N* is the number of reactions.

Let us assume that there are *N*_*E*_ fast/equilibrium reactions (all of which must be independent) and *N*_*K*_ slow/kinetic reactions, i.e., *N* = *N*_*E*_ + *N*_*K*_.

For an elementary kinetic reaction, the rate law is given by collision theory as

7 $$R_{K} = K_{K}^{f} \prod\limits_{i = 1}^{M} {\left( {A_{i} } \right)^{{\mu_{ik} }} } - K_{K}^{b} \prod\limits_{i = 1}^{M} {\left( {A_{i} } \right)^{{\nu_{ik} }} } \varvec{,}\quad K \in \varvec{N}_{K}$$

where *R*_*k*_ is the rate of the *k*th kinetic reaction, *A*_*i*_ is the activity of the *i*th species, $K_{K}^{f}$ and $K_{K}^{b}$ are the activity-based forward and backward rate constant of the *k*th kinetic reaction, respectively, and *N*_*K*_ is the number of kinetic reactions. The forward and backward rate constants cannot be determined sequentially with the measurement of concentration-versus-time curves of all species because *N*_*K*_ equations in Eq. (7) are coupled regarding the forward and backward rate constants.

If the reaction is an equilibrium reaction, the reaction rate is infinity, which results in the law of mass action as:

8 $$R_{K} = \infty \;\exists \;K_{K}^{e} = {{\left( {\prod\limits_{i = 1}^{M} {\left( {A_{i} } \right)^{{\mu_{ik} }} } } \right)} \mathord{\left/ {\vphantom {{\left( {\prod\limits_{i = 1}^{M} {\left( {A_{i} } \right)^{{\mu_{ik} }} } } \right)} {\left( {\prod\limits_{i = 1}^{M} {\left( {A_{i} } \right)^{{\nu_{ik} }} } } \right)}}} \right. \kern-0pt} {\left( {\prod\limits_{i = 1}^{M} {\left( {A_{i} } \right)^{{\nu_{ik} }} } } \right)}}\varvec{,}\quad K \in N_{E}$$

where $K_{K}^{e}$ is the equilibrium constant of the *k*th reaction, *A*_*i*_ is the activities of the *i*th species, and *N*_*E*_ is the number of linearly independent equilibrium reactions. The equilibrium constants may be determined sequentially with the measurement of the activities of all species.

#### Effect of precipitation/dissolution on porosity, hydraulic conductivity, water capacity and hydrodynamic dispersion

The effective moisture content, particularly in terms of the pressure head (by the degree of saturation S), and the concentrations of geochemical species may be theoretically derived as

9 $$\theta = \frac{{S\theta_{so} }}{{1 + S\varphi_{p} }};\quad \varphi_{p} = \varSigma P_{i} V_{i}$$

where *θ* is the effective moisture content [dm^3^ of water/dm^3^ of pore]; *θ*_*so*_ is the effective saturated moisture content when solid or surface biogeochemical species are not present [dm^3^ of pores/dm^3^ of medium]; *φ*_*p*_ is the volume precipitated species per unit volume of water [dm^3^ of precipitates/dm^3^ of water]; *P*_*i*_ is the precipitated concentration of the *i*th mineral [mol/dm^3^ of water]; *V*_*i*_ is the mole volume of the *i*th mineral [dm^3^ of mineral/mole of mineral]; and *i* represents the *i*th precipitated mineral. The hydraulic conductivity and the water capacity in Eq. (9) were modified to incorporate the effect of precipitation–dissolution as the first and second equations, respectively, in Eq. (10), as follows:

10 $$K = \frac{{\left( {\frac{\rho }{{\rho_{o} }}} \right)}}{{\frac{\mu }{{\mu_{o} }}}}K_{so} k_{r} \left( {\frac{1}{{1 + S\varphi_{p} }}} \right)^{n} ;\quad \psi = \frac{{\theta_{so} }}{{1 + S\varphi_{p} }}\frac{dS}{dh}$$

where *n* is the fractional exponent, depending on particle size and packing structure, and $\psi \equiv n_{e} \cdot \frac{dS}{dh}$ is the water capacity.

The effect of precipitation/dissolution on hydrodynamic dispersion was derived as (Yeh et al. 2001, 2004)

11 $$\theta D = \left[ {\alpha_{T} \left| V \right|\delta + \left( {\alpha_{L} - \alpha_{T} } \right)VV\left| V \right| + \theta D_{w} \tau \delta } \right]\left[ {\theta^{m - 1} \left( {1 - \varphi_{p} } \right)^{m} } \right]$$

where *α*_*L*_ and *α*_*T*_ are the longitudinal and transverse diffusivity, respectively [L]; *δ* is Kronecker delta tensor; |*V*| is the magnitude of the Darcy’s velocity [L/T]; *D*_*w*_ is the molecular diffusion coefficient of pure water [L^2^/T]; *τ* is tortuosity; *m* is the cementation exponent (Dullien 1979) with reported values between 1.3 and 2.5; and *θ* is either the input moisture content or equal to the moisture content, as calculated in the coupled flow simulation.

#### Numerical method

A two-step method was used to solve the chemical transport equations and chemical equilibrium equations. Once the solution for one time step converges, the calculation continues to the next time step. The finite difference (FD) methods were used for temporal discretization of the governing partial differential equations in the flow module and reactive transport module. The Galerkin finite element method was used for spatial discretization of the modified Richards equation that governs the distribution of pressure fields. For scalar reactive transport equations, either the conventional finite element methods or the hybrid Lagrangian–Eulerian finite element methods were used for spatial discretization. The chemical equilibrium equations were solved by the Newton–Raphson method or Picard method.

## Simulation of concrete degradation

### Thermodynamic data and PHREEQCI modeling

The composition and phase development of hydration products as the chemical reaction of cement with groundwater flow influences the lifetime performance of the concrete barrier. The thermodynamic modeling of Portland cement in the subsurface system of cementitious media was formulated and applied to assess the performance of concrete barriers.

Blanc et al. (2010a, b) established the thermodynamic data of the chemical model for the phases of system CaO–SiO_2_–H_2_O, CaO–Al_2_O_3_–SiO_2_–H_2_O, CaO–Al_2_O_3_–SO_3_–CO_2_–Cl–H_2_O in the water and cement-based materials. In addition, Galíndez and Molinero (2010) used the reactive transport model to simulate the degradation of cementitious materials. These thermodynamic data allow the reactive transport model to simulate the composition and concentration of chemical species under various hydrogeochemical environments. Table 3 lists the thermodynamic data that is associated with the described reactions of concrete degradation in a water–solid system of this study.

**Table 3:** Reactions considered in the HYDROGEOCHEM 5.0 model

Aqueous complexation reactions (25 °C)					
No.	Reaction	log K	No.	Reaction	log K
1	H_2_O = OH^−^ + H^+^	−14	20	K^+^ + Cl^−^ = KCl	−1.49
2	Al^3+^ + 4OH^−^ = AlO_2_^−^ + 2H_2_O	33.12	21	K^+^ + OH^−^ = KOH	−0.46
3	Al(OH)_2_+ = Al^3+^ + 2(OH)^−^	−3.41	22	K^+^ + SO_4_^2−^ = KSO_4_^−^	0.85
4	Al(OH)^2+^ = Al^3+^ + (OH)^−^	−9.05	23	NaAlO_2_ + 2H_2_O = Al^3+^ + Na^+^ + 4OH^−^	−18.37
5	Al(SO_4_)_2_^−^ = Al^3+^ + 2SO_4_^2−^	−4.9	24	Na^+^ + Cl^−^ = NaCl	−0.78
6	AlSO_2_^+^ = Al^3+^ + SO_4_^2−^	−3.01	25	NaHCO_3_ = HCO_3_^−^ + Na^+^	−0.15
7	Ca^+2^ + HCO_3_^−^ + OH^−^ = CaCO_3_ + H_2_O	6.895	26	Na^+^ + H_4_SiO_4_ + OH^−^ = NaH_3_SiO_4_(aq) + H_2_O	5.99
8	Ca^2+^ + Cl^−^ = CaCl^+^	−0.7	27	Na^+^ + HCO_3_^−^ + OH^−^ = NaCO_3_^−^ + H_2_O	4.19
9	CaCl_2_ = Ca^2+^ + 2Cl^−^	0.64	28	Na^+^ + OH^−^ = NaOH	−0.79
10	CaHCO_3_^+^ = Ca^2+^ + HCO_3_^−^	−1.05	29	Na^+^ + SO_4_^2−^ = NaSO_4_^−^	0.82
11	Ca^2+^ + OH^−^ = CaOH^+^	−1.15	30	HCO_3_^−^ + 9 H^+^ + 8 e^−^ = CH_4_(aq) + 3 H_2_O	30.742
12	Ca^2+^ + SO_4_^2−^ = CaSO_4_	2.11	31	2 H_2_O = O_2_(aq) + 4 H^+^ + 4 e^−^	−86.08
13	HCO_3_^−^ = CO_2_(aq) + OH^−^	7.66	32	2 H^+^ + 2 e^−^ = H_2_(aq)	−3.15
14	HCO_3_^−^ + OH^−^ = CO_3_^2−^ + H2O	−3.67	33	SO_4_^2−^ + 8 H^+^ + 8 e^−^ = S^2−^ + 4 H_2_O	20.732
15	HAlO_2_(aq) + H_2_O = Al^3+^ + 3(OH)^−^	−25.57	34	SO_4_^2−^ + 9 H^+^ + 8 e^−^ = HS^−^ + 4 H_2_O	33.65
16	HCl +OH^−^ = Cl^−^ + H_2_O	13.33	35	SO_4_^2−^ + 10 H^+^ + 8 e^−^ = H_2_S + 4 H_2_O	40.644
17	H_4_SiO_4_ + 2OH^−^ = H_2_SiO_4_^2−^ + 2H_2_O	5	36	Fe^+3^ + e^−^ = Fe^+2^	13.02
18	H_2_SO_4_ + 2(OH)^−^ = SO_4_^2−^ + 2H_2_O	29.02	37	Fe^+3^ + 4 H_2_O = Fe(OH)_4_^−^ + 4 H^+^	−21.6
19	HSO_4_^−^ + OH^−^ = H_2_O + SO_4_^2−^	12.02			

Precipitation-dissolution reactions (25 °C)				
No.	Mineral	Reaction	log K	Molar volume (dm^3^/mol)
1	Calcite	HCO_3_^−^ + Ca^2+^ + OH^−^ = CaCO_3_ + H_2_O	12.151	0.03693
2	Portlandite	Ca^2+^ + 2OH^−^ = Ca(OH)_2_	5.20	0.03300
3	Ettringite	2Al^3+^ + 6Ca^2+^ + 26H_2_O + 3SO_4_^2−^ + 12OH^−^ = Ca_6_Al_2_(SO_4_)_3_(OH)_12_·26H_2_O	111.03	0.71032
4	Quartz	H_4_SiO_4_ = SiO_2_ + 2H_2_O	3.98	0.02269
5	Gypsum	Ca^2+^ + SO_4_^2−^ + 2H_2_O = CaSO_4_·2H_2_O	4.58	0.07470
6	Hydrogarnet	2Al^3+^ + 3Ca^2+^ + 12OH^−^ = Ca_3_Al_2_(OH)_12_	87.68	0.14952
7	Friedel’s salt	2Al^3+^ + 4Ca^2+^ + 2Cl^−^ + 4H_2_O + 12OH^−^ = 2Ca_2_Al(OH)_6_Cl·2H_2_O	93.07	0.27624
8	Thaumasite	3Ca^2+^+H_4_SiO_4_ + SO_4_^2−^+HCO_3_^−^+11H_2_O +3OH^−^ = CaSiO_3_·CaSO_4_·CaCO_3_·15H_2_O	31.70	0.32940
9	Monocarboaluminate	2Al^2+^ +HCO_3_^−^ + 4Ca^2+^ + 3.68H_2_O + 13OH^−^ = 3CaO·Al_2_O_3_·CaCO_3_·10.68H_2_O	101.45	0.26196
10	Pyrite	FeS_2_ + 2 H^+^ + 2 e^−^ = Fe^+2^ + 2 HS^−^	−18.479	0.02394

The PHREEQCI was first used to calculate the porewater composition that equilibrated with rainwater (see Table 4), groundwater, and geological mineral of argillite (that is, montmorillonite, calcite, chlorite, dolomite, illite, feldspar, mica, kaolinite, pyrite, quartz, and siderite).

**Table 4:** Rain water quality used in the thermodynamic equilibrium model

Species	Cl^−^	SO_4_^2−^	Na^+^	K^+^	Ca^2+^	Mg^2+^	pH
Concentration (Units: μmol/l)	158.2	59.5	84.8	64.5	19	25.3	5.2

### HYDROGEOCHEM 5.0 simulation

**Fig. 3:**
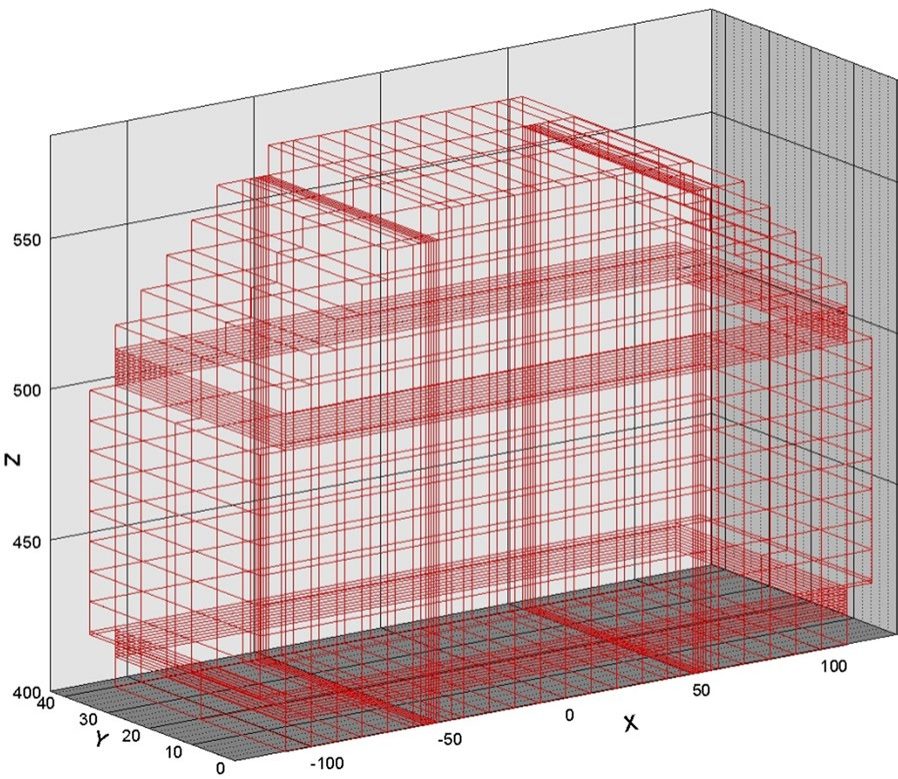
The numerical discretization of grids for HYDROGEOCHEM 5.0

**Fig. 4:**
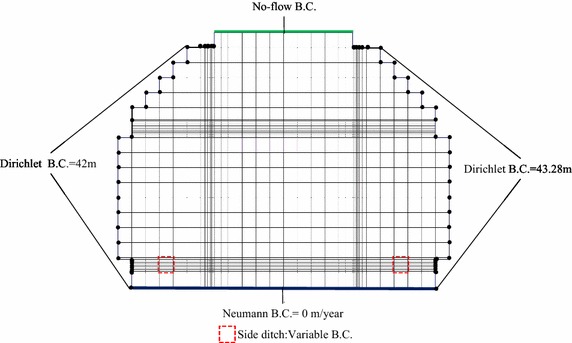
Boundary conditions for groundwater flow simulation of HYDROGEOCHEM 5.0

The simulation region with an elevation of 40 m at the bottom was discretized with 3918 elements and 4146 nodes. The grid comprised cells of dimensions Δx, the Δy varied from 1 to 0.1 m, and Δz = 2 m, as illustrated in Fig. 3. The vertical front edge, back edge, and horizontal top edge depict a no-flow boundary, except for the side ditch in the vertical front edge. The side ditch area on the front edge was set to a variable boundary condition, which is usually an air-media interface in which water uninterruptedly seeps out. The flux in the bottom edge caused by gravity was considered a Neumann boundary with a zero flux. The horizontal left and right edges were Dirichlet head-boundary conditions. This hydraulic head value of Dirichlet boundary was estimated from the steady-state simulated groundwater flow (that is, Vx = 4.0 × 10^−8^ m/s, to left; Vz = 3.3 × 10^−8^ m/s, downward) in the proposed site of Arnold et al. (2007). The estimated total head on the horizontal left and right edge nodes were assumed as 420 and 432.8 dm, respectively (Fig. 4).

**Fig. 5:**
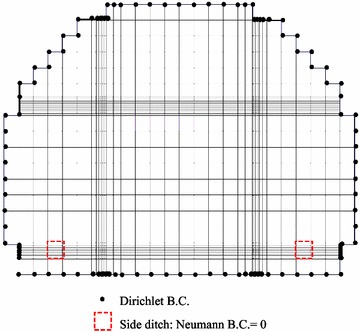
Boundary conditions for reactive transport simulation of HYDROGEOCHEM 5.0

The cementitious materials of the concrete of the EBS may interact with the groundwater when the repository begins to operate. It is crucial to avoid the concrete immersion by the inflow of groundwater to mitigate this type of interaction. The Taiwan Power Company proposed a side ditch design to install at the bottom of the disposal tunnel and allow the drainage of inflow water. If the groundwater inflow is drained from the side ditch, the effect of concrete degradation on the performance of EBS may be reduced. Hence, the simulation scenarios considered in this study were the performance assessment of EBS with and without the side ditch. Table 5 and Figs. 4, 5 provide a summary of the physical parameters for groundwater flow and the reactive transport simulations. The study considered the hydrogeochemical transport of 11 components, as follows: Na^+^, K^+^, Ca^2+^, Al^3+^, OH^−^, HCO_3_^−^, Cl^−^, SO_4_^2−^, H_4_SiO_4_, Fe^3+^, e^−^, 37 aqueous species, and 10 minerals of precipitation/dissolution reactions. The model simulation time was 300 years with an initial time-step of 1.0 × 10^−5^ year, and a maximal allowable time-step of 0.01 year. In addition, the precipitation/dissolution on the change of porosity, hydraulic conductivity, and hydrodynamic dispersion of cementitious media may be a crucial hydrogeochemical mechanism in the concrete degradation. Thus, the effect of precipitation/dissolution reactions on both the flow and the reactive transport were also considered in the simulations. Moreover, pyrite oxidation and sulfate reduction reactions were also incorporated in the simulation scenarios to evaluate the effect of redox processes on the formations of degradation materials. Table 6 lists the initial and boundary conditions, and other parameters that were used in this study for reactive chemical transport modeling.

**Table 5:** Physical parameters used in HYDROGEOCHEM 5.0 model

Media	K (m/s)	Porosity (%)	Diffusion coefficient (m^2^/s)	Bulk density (kg/m^3^)	Longitudinal dispersivity (m)	Lateral dispersivity (m)
Concrete	3.1 × 10^−14^	0.15	3.0 × 10^−12^	2030	0.10	0.010
Bentonite	5.0 × 10^−11^	0.363	1.2 × 10^−10^	2000	0.15	0.015
Backfill	3.24 × 10^−8^	0.168	2.0 × 10^−11^	2410	0.30	0.030

**Table 6:** Initial and boundary conditions of component concentration (mol/l) used in HYDROGEOCHEM 5.0 model. Source: ^a^Taiwan Cement Company (2011); ^b^Arcos et al. (2006); ^c^Taiwan Power Company (2010)

Component	Initial conditions			Boundary conditions
	Concrete^a^	Bentonite^b^	Backfill^c^	Groundwater
Na^+^	6.908 × 10^−2^	1.69 × 10^−1^	1.01 × 10^−4^	7.68 × 10^−5^
K^+^	0.2665	1.14 × 10^−3^	1.84 × 10^−6^	1.84 × 10^−6^
Ca^2+^	29.906	9.97 × 10^−3^	3.61 × 10^−2^	3.21 × 10^−2^
Al^3+^	2.628	1.00 × 10^−20^	3.33 × 10^−14^	2.71 × 10^−8^
OH^−^	21.1	1.91 × 10^−7^	3.43 × 10^−7^	3.43 × 10^−7^
HCO_3_^−^	1.394 × 10^−11^	2.14 × 10^−3^	3.60 × 10^−4^	3.81 × 10^−4^
Cl^−^	5.64 × 10^−5^	1.53 × 10^−1^	1.79 × 10^−4^	1.58 × 10^−4^
SO_4_^2−^	1.633 × 10^−3^	2.94 × 10^−2^	8.57 × 10^−2^	8.81 × 10^−2^
H_4_SiO_4_	9.198	6.60 × 10^−5^	1.51 × 10^−4^	1.51 × 10^−4^
Fe^3+^	3.10 × 10^−2^	1.0 × 10^−8^	1.0 × 10^−10^	8.79 × 10^−4^
pe	−1.0	−0.70	−1.0	−1.0

Three cases of reactive transport simulations were considered, as follows:

- Case 1: LLW repository without side ditch and redox processes.
- Case 2: LLW repository with side ditch and without redox processes.
- Case 3: LLW repository with side ditch and redox processes.

## Results and discussion

**Fig. 6:**
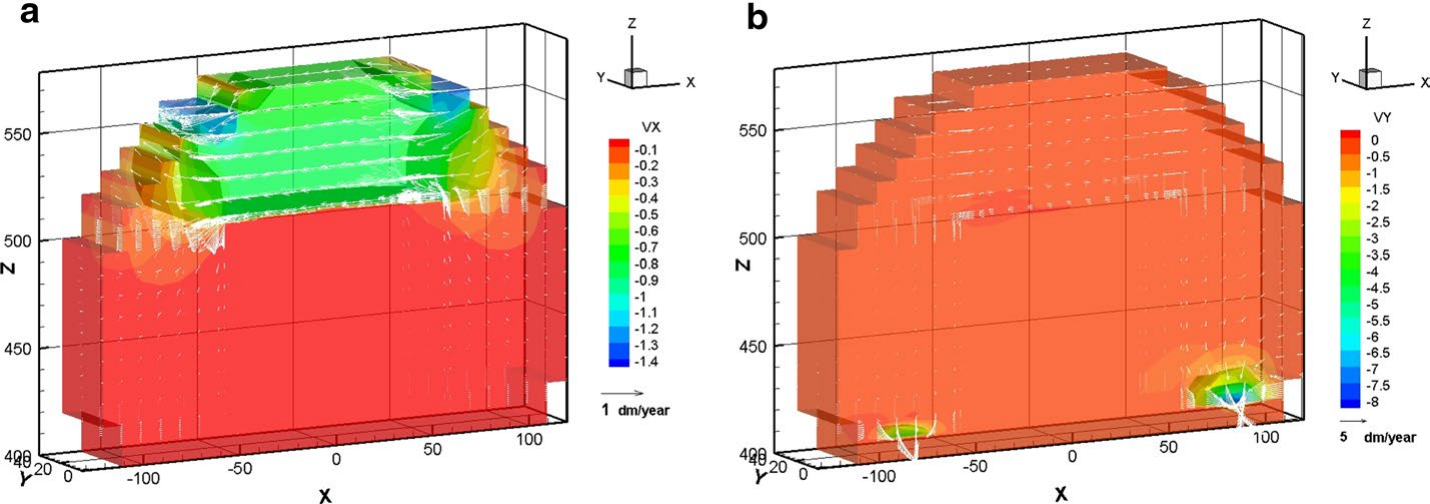
Velocity distribution of steady state flow for Case 1 (**a**), Case 2 (**b**), and Case 3 (**b**)

The simulations of Cases 1, 2, and 3 were conducted by using HYDROGEOCHEM 5.0. The simulated results revealed notable differences in the concrete degradation. Figure 6 illustrates the steady state distribution of the flow velocity of Cases 1, 2, and 3. The high groundwater inflow rate was developed quickly and drained out to the side ditch in Case 2 and Case 3. The highest groundwater velocity of Cases 2 and 3 was 5.7 times greater than that of Case 1.

**Fig. 7:**
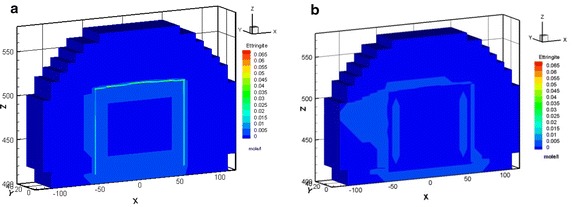
Ettringite distribution for Case 1 (**a**) and Case 2 (**b**) after 300 years

In Case 1, the ettringite was formed with a maximal amount of approximately 2.49 × 10^−2^ mol/l at the vertical left, right, and horizontal top edge areas of the concrete barrier within 100 years. After 300 years, as illustrated in Fig. 7a, the amount of precipitated ettringite increased with time, and reached a maximal value of 7.01 × 10^−2^ M because of the continuous sulfate intrusion. However, the ettringite was mildly formed in Case 2, with an amount of 1.53 × 10^−4^ M precipitated at the top right edge of the concrete barrier after 300 years (Fig. 7b).

**Fig. 8:**
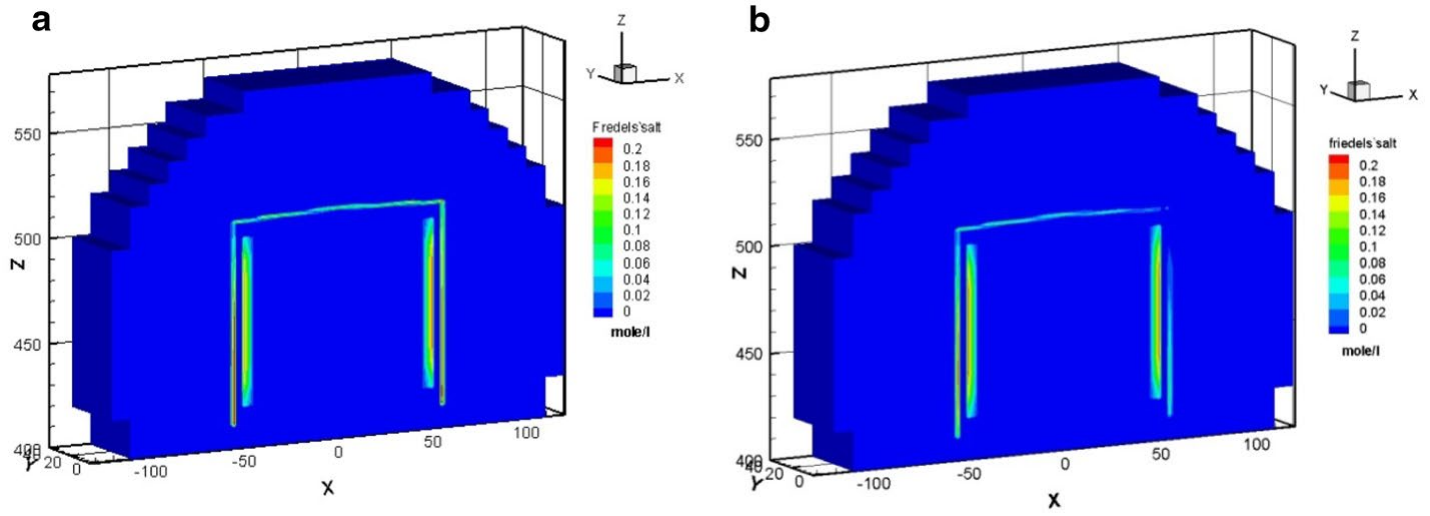
Friedel’s salt distribution for Case 1 (**a**) and Case 2 (**b**) after 300 years

**Fig. 9:**
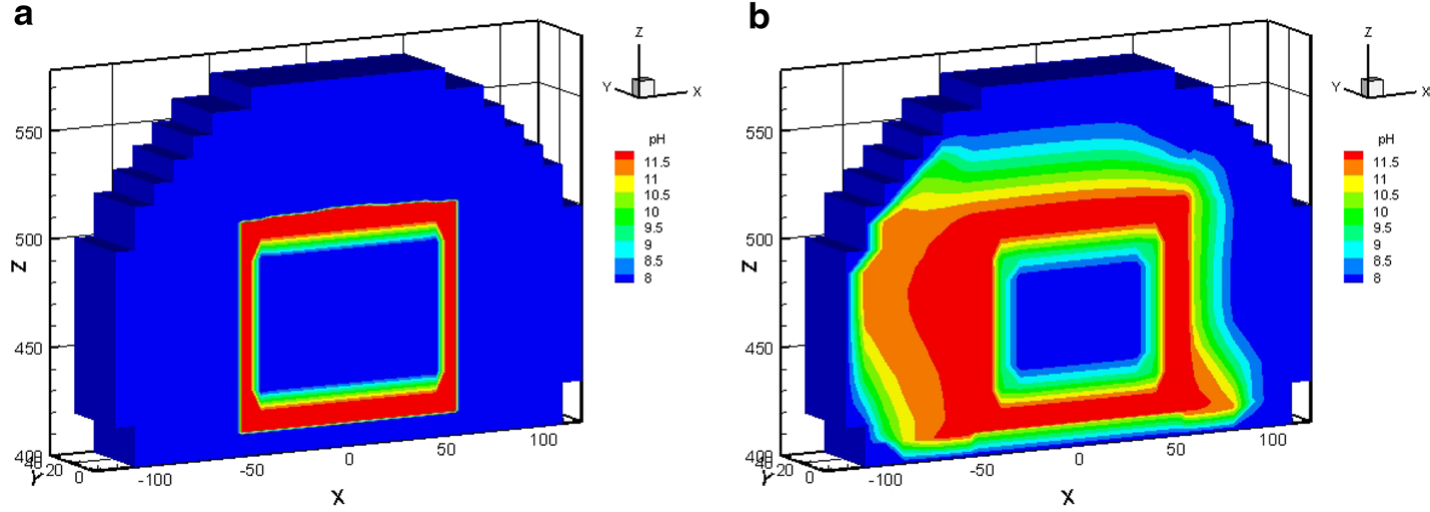
pH distribution for Case 2 in 0 years (**a**) and after 300 years (**b**)

**Fig. 10:**
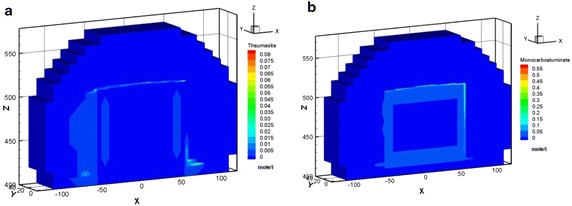
Distribution of thaumasite (**a**) and monocarboaluminate (**b**) for Case 2 after 300 years

**Fig. 11:**
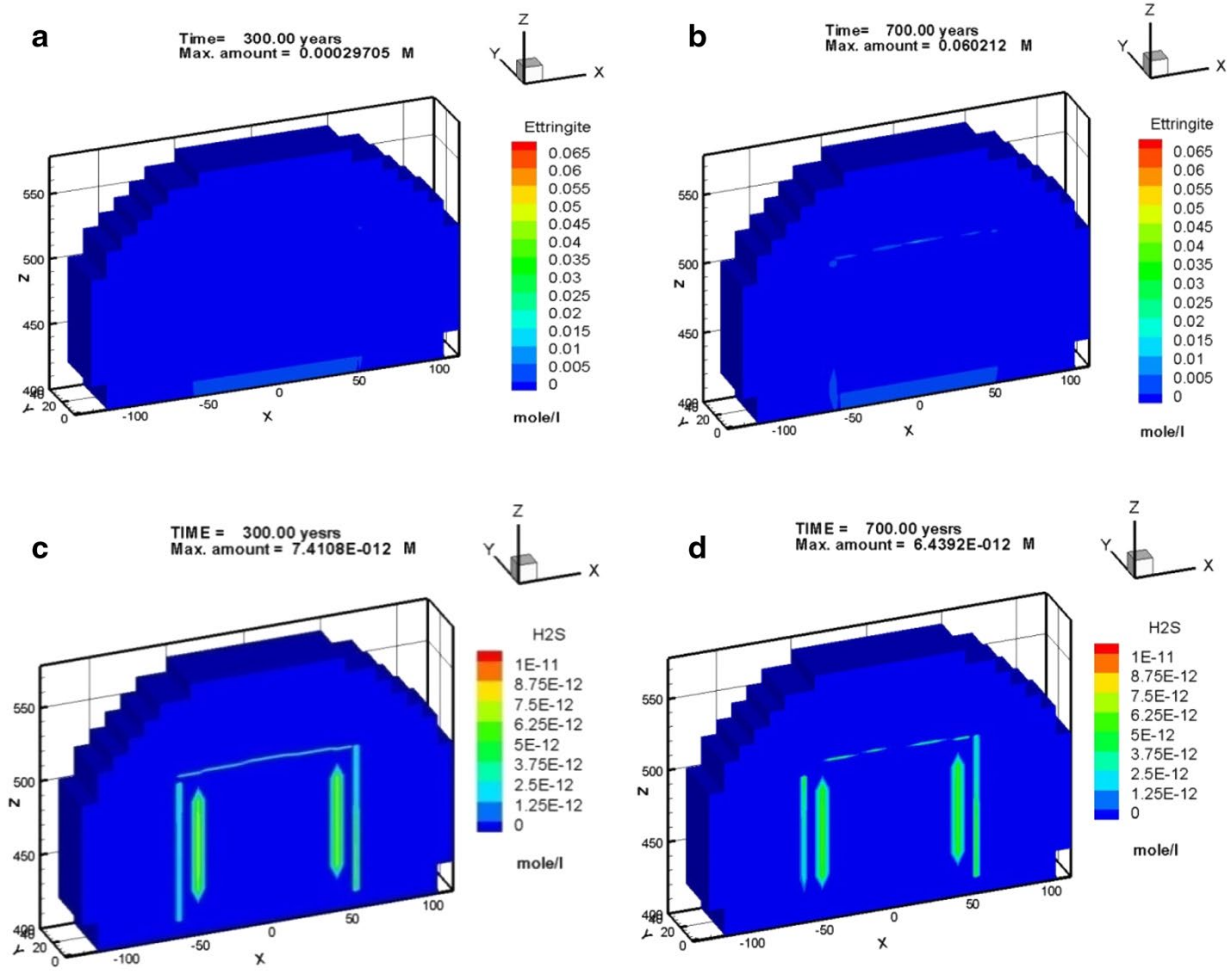
Distribution of ettringite and hydrogen sulfide for Case 3 after 300 and 700 years: **a** ettringite 300 years, **b** ettringite 700 years, **c** hydrogen sulfide 300 years, and **d** hydrogen sulfide 700 years

In Case 1, the maximal amount was approximately 2.94 × 10^−1^ mol/l of Friedel’s salt formed at the vertical left, right edge and horizontal top edge areas of the concrete barrier within 100 years. The amount of Friedel’s salt increases as time progress reaching a maximal value of approximately 2.70 × 10^−1^ M in 300 years, as illustrated in Fig. 8a. Nevertheless, in Case 2, Friedel’s salt moderately formed with an of amount of 2.23 × 10^−1^ M at the vertical left edge of the concrete barrier after 300 years, as shown in Fig. 8b. In addition, Friedel’s salt depleted in the area of the top right edge. The amounts of portlandite that was precipitated in cases 1 and 2 were similar. The portlandite formed over the entire concrete barrier in less than 300 years. The portlandite was consistently stable. In Case 2, the pH undergoes a small change due to the interaction between the groundwater and the cementitious minerals in the concrete barrier. Initially, the pH was close to 12, as the simulation proceeded, the hydrogen ion plume intruded and the pH subsequently decreased to 11.5 after 300 years, as shown in Fig. 9. This process also results a small depletion of calcium in the cementitious minerals of concrete barrier. The amount and distribution of hydrogen ion, sulfate, and chloride species in the EBS showed significant differences in Case 1 and Case 2. In Case 1 the amount of these species was one order of magnitude higher than that of in Case 2. Thaumasite and monocarboaluminate also formed as carbonate aggregates by sulfate attack and carbonate intrusion, respectively (see Fig. 10). Sufficient evidences show that the main processes that were responsible for concrete degradation are the species induced from hydrogen ion, sulfate, and chloride. Concentrations of the hydrogen ion, sulfate, and chloride species in the EBS are affected by the rate of advective transport of groundwater outflow to the side ditch drainage system. Concentrations of hydrogen ion, sulfate, and chloride in the EBS with the side ditch are lower than that of the EBS without the side ditch. The EBS with side ditch is efficient to drain the groundwater and lowers the concentration of the species induced concrete degradation in 300 years. Thus the EBS with the side ditch can divert the water and reduce the concrete degradation. In addition, the ettringite was slightly formed in Case 3 with a maximum amount of 2.86 × 10^−4^ M precipitated at top right edge of the concrete barrier after 300 years (Fig. 11a). As the simulation proceeds, the region of ettringite formation in Case 3 after 700 years was still much less than that of in Case 2 after 300 years (Fig. 11b). In Case 3, the redox processes controlled the geochemical reactions, such as pyrite oxidation, and sulfate reduction concomitantly occurred in the concrete degradation processes. Appelo and Postma (2005) pointed out that the ongoing sulfate reduction may be recognized by the presence of H_2_S in the groundwater. Figures 11c and d show that H_2_S was formed at the left, top, and right edge of the concrete barrier. Thus, the sulfate reduction can reduce the concentration of sulfate and ettringite formation. Therefore, the redox processes significantly influence on the formation of degradation materials. Reduction environment in the EBS can reduce the ettringite formation in the concrete degradation processes.

This study considered limited chemical reactions under the chemical equilibrium condition; therefore, the comprehensive reactions of the cementitious phase and the chemical kinetics should be included in future reactive chemical transport simulations. Based on the simulated results, the hydrogen ion can result in the dissolution of portlandite and increases the concentration of calcium in the pore water. The sulfate anion reacts with cement to form ettringite. The chloride from the pore water enters the cementitious materials to form Friedel’s salt. Luna et al. (2006) pointed out that the ettringite can cause the volumetric expansion and eventually leads to fracturation processes. Excess of chloride can produce corrosion of the reinforcement element and cause a volume expansion which may lead to micro-fracturing in the concrete. Formation of ettringite and Friedel’s salt are also associated to a volume increase eventually leading to fracture formation and weaken the EBS (Lagerblad and Trägardh 1994). Seitz and Walton (1993) showed the detailed failure mechanisms for concrete vaults. They pointed out that, as cracks fully penetrate the concrete, the permeability of the vault increases. The cracks can also accelerate degradation rates. Thus, the water flow through cracks over extended periods is the primary concern for concrete performance of long life radionuclides (Seitz and Walton 1993). Moreover, seismic fractures may induce the coincided cracks and increase permeability. However, HYDROGEOCHEM 5.0 is not able to predict crack damage or its impact on permeability. The further quantification of concrete degradation caused by concrete cracks and numerical models coupled with thermo-hydraulic–mechanical–chemical processes should be developed to assess these impacts in the future.

## Conclusions

A proposed site for final disposal of LLW of Daren is on the selected list in Taiwan. To investigate the hydrogeochemical reactions and effect on concrete degradation in the proposed LLW repository site, HYDROGEOCHEM 5.0 model was applied to simulate the complex chemical interactions between the cement minerals of the concrete and groundwater flow and reactive chemical transport resulting from water–rock interaction in the proposed site. Simulation results show the main processes responsible for concrete degradation involve geochemical reactive species induced from hydrogen ion, sulfate, and chloride. The intrusion of hydrogen ion from groundwater results the dissolution of portlandite. However, the portlandite still maintains alkaline condition in proposed LLW repository, the concrete used as a confinement material in EBS may remain in well durable condition. The sulfate-induced concrete degradation may form the ettringite and the chloride-induced concrete degradation form Friedel’s salt. Thaumasite and monocarboaluminate are also formed as carbonate aggregates by sulfate attack, and carbonate intrusion, respectively. The formation of ettringite, Friedel’s salt, thaumasite and monocarboaluminate may result a volume expansion. The EBS with the side ditch efficiently drains the ground water and lowers the concentration of concrete degradation induced species. The degree of concrete degradation on performance of EBS with side ditch is much lower than the disposal tunnel without the side ditch. Moreover, the redox processes significantly influence on the formations of degradation materials. Reductive environment in the EBS can reduce the ettringite formation in the concrete degradation processes. The results of the study provide a detail picture of the long-term evolution of the hydrogeochemical environment of the proposed LLW disposal site in Taiwan. The chemical kinetics effect should be included in future reactive chemical transport simulations. Moreover, the interaction among water and other minerals of cement, bentonite, and backfill materials in the near field, volume-expanded cracks and seismic induced-fractures should also be considered in the future research. The development of advanced numerical models that coupled with thermo-hydraulic–mechanical–chemical processes is especially welcomed.
